# Secretoneurin A regulates neurogenic and inflammatory transcriptional networks in goldfish (*Carassius auratus*) radial glia

**DOI:** 10.1038/s41598-017-14930-8

**Published:** 2017-11-02

**Authors:** Dillon F. Da Fonte, Christopher J. Martyniuk, Lei Xing, Adrian Pelin, Nicolas Corradi, Wei Hu, Vance L. Trudeau

**Affiliations:** 1 Department of Biology, University of Ottawa, Ontario, K1N 6N5 Canada; 2 Department of Physiological Sciences and Center for Environmental and Human Toxicology, University of Florida Genetics Institute, Interdisciplinary Program in Biomedical Sciences Neuroscience, College of Veterinary Medicine, University of Florida, Gainesville, FL 32611 USA; 3 State Key Laboratory of Freshwater Ecology and Biotechnology, Institute of Hydrobiology, Chinese Academy of Sciences, Wuhan, 430072 China; 4 Present Address: Max Planck Institute of Molecular Cell Biology and Genetics, Dresden, 01307 Germany

## Abstract

Radial glial cells (RGCs) are the most abundant macroglia in the teleost brain and have established roles in neurogenesis and neurosteroidogenesis; however, their transcriptome remains uncharacterized, which limits functional understanding of this important cell type. Using cultured goldfish RGCs, RNA sequencing and *de novo* transcriptome assembly were performed, generating the first reference transcriptome for fish RGCs with 17,620 unique genes identified. These data revealed that RGCs express a diverse repertoire of receptors and signaling molecules, suggesting that RGCs may respond to and synthesize an array of hormones, peptides, cytokines, and growth factors. Building upon neuroanatomical data and studies investigating direct neuronal regulation of RGC physiology, differential gene expression analysis was conducted to identify transcriptional networks that are responsive to the conserved secretogranin II-derived neuropeptide secretoneurin A (SNa). Pathway analysis of the transcriptome indicated that cellular processes related to the central nervous system (e.g., neurogenesis, synaptic plasticity, glial cell development) and immune functions (e.g., immune system activation, leukocyte function, macrophage response) were preferentially modulated by SNa. These data reveal an array of new functions that are proposed to be critical to neuronal-glial interactions through the mediator SNa.

## Introduction

Radial glial cells (RGCs) are a progenitor subtype in the developing central nervous system (CNS)^1^ and have a bipolar morphology with radial fibers that serve as scaffolds for neuronal migration^2^. RGCs are self-renewing through proliferative symmetrical divisions and are multipotent, generating neurons or other glial cells^3^. In mammals, RGCs are mostly a transient cell type, differentiating into neurons and glia at the end of development. They only persist in two areas of the adult brain, the anterior part of the subventricular zone of the lateral ventricle and subgranular zone of the dentate gyrus, where they serve as progenitor cells. These two areas are the main constitutive neurogenic regions of the adult mammalian brain, which explains the limited capacity for adult neurogenesis in mammals^4,5^. In contrast, RGCs are abundant throughout teleost development and into adulthood in numerous brain neurogenic zones^6^, which may explain why teleost fish exhibit the most pronounced and widespread adult neurogenesis of any vertebrate taxon studied thus far^7,8^. In addition to being a progenitor subpopulation, RGCs are also the only macroglia in teleost fish, as they lack bona fide stellate astrocytes^9^.

Apart from studies that have established their function in producing neuroestrogens through the expression and function of the steroidogenic enzyme aromatase B^10–12^, little is known about other functions performed by RGCs and the regulatory factors that control this cell type to maintain brain homeostasis^13^. While transcriptomics have been used recently to reveal the diversity of human radial glia^14,15^, fish RGCs remain uncharacterized at the transcriptomic level.

Given that RGCs make direct contact with cerebrospinal fluid and with adjacent neurons, they have the potential to be under regulation by numerous signaling molecules, such as hormones, neurotransmitters, neuropeptides, and cytokines. Previous reports in fish have identified dopaminergic^16–18^ and serotonergic regulation^19^ of RGC physiology. Expanding on these neuronal-RGC interactions, our recent study highlighted the neuroanatomical relationship between RGCs and soma of magnocellular and parvocellular neurons immunoreactive for the secretogranin II (SgII)-derived neuropeptide secretoneurin A (SNa) in the goldfish preoptic nucleus^20^. SN is an evolutionary conserved neuropeptide generated by endoproteolytic processing of its precursor protein SgII and is found in dense-core secretory granules in a wide variety of cell types of the endocrine and nervous systems^21–23^. SgII belongs to the chromogranin family that have a high proportion of acidic amino acids, the capacity to bind calcium, and the ability to form aggregates at low pH levels^24^. Teleost fish have two SgII paralogs, SgIIa and SgIIb, likely generated by the whole genome duplication event that occurred in the teleost lineage^23^.

SN exerts a diverse array of biological functions and controls nervous, endocrine, immune, and vascular systems^22,25,26^, reflecting the wide distribution of the peptide in the body. In the immune system, SN exerts chemotactic effects on several types of immune cells such as monocytes, eosinophils, natural killer, and endothelial cells^26^. Moreover, in the CNS, SN acts as a trophic factor stimulating neurite outgrowth^27^, assists in the growth and repair of neuronal tissue by promoting neuroprotection and plasticity^28^ and regulates the release of several key neurotransmitters such as glutamate, dynorphin B, dopamine, and γ-aminobutyric acid (GABA)^29–31^. In addition to its role in these physiological processes, SN is also implicated in many pathophysiological conditions of the CNS including, brain ischemia^28,32^, Alzheimer disease^33,34^, and epilepsy^35^. Although SN has many attributed functions in both the periphery and CNS, nothing is known about how this peptide regulates glia. Therefore, the objective of this study was to generate the first reference RGC transcriptome for fish in order to identify new putative functions that are critical to neuronal-RGC communication through SNa.

## Materials and Methods

### Experimental animals

All procedures were approved by the University of Ottawa Protocol Review Committee and followed standard Canadian Council on Animal Care guidelines on the use of animals in research. Common adult female goldfish *(Carassius auratus)* were purchased from a commercial supplier (Mt. Parnell Fisheries Inc.) and allowed to acclimate for at least 3 weeks prior to experimentation. Animals were maintained at 18 °C under a natural stimulated photoperiod and fed standard flaked goldfish food. Sexually mature female goldfish (18–35 g) were anesthetized using 3-aminobenzoic acid ethylester (MS222) for all handling and dissection procedures.

### Cell culture and exposure

Cell culture methods have been previously established and validated for RGCs^16^. Briefly, the hypothalamus and telencephalon were dissected from female goldfish and rinsed twice with Hanks Balanced Salt Solution (HBSS; 400 mg KCl, 600 mg KH_2_PO_4_, 350 mg NaHCO_2_, 8 g NaCl, 48 mg Na_2_HPO_4_, and 1 g D-Glucose in 1 L ddH2O) with Antibiotic-Antimycotic solution (Gibco) and minced into small explants. RGCs were dissociated with trypsin (0.25%; Gibco) and cultured in Leibovitz’s L-15 medium (Gibco) with 15% Fetal Bovine Serum (FBS; Gibco) and Antibiotic-Antimycotic (Gibco). Cell culture medium was changed 4–7 days after isolation and then once a week thereafter. RGCs were subcultured by trypsinization (0.125%) for 3 passages, and then were used for experimentation. This results in RGC cultures in which >95% of the cells co-express glial fibrillary acid protein and brain lipid binding protein^16^. The focus of our study was on goldfish SNa because it is the only fish SN with known biological activity, and we have described the neuroanatomical relationship between RGCs and SNa-positive neurons^20^. Goldfish SNa was synthesized as reported^36^. Stock solutions of purified goldfish SNa peptide were made in distilled sterile water and stored at −20 °C until use. Aliquots were thawed on ice then diluted to desired concentrations in serum-free media. Previous studies have shown SN stimulates monocyte migration in a range of 0.01 to 1000 nM^37,38^ and increases neurite outgrowth at concentrations between 50–300 nM^27^. Here, we aimed to characterize the response to a maximal dose of SNa, so passage 3 RGCs were exposed to 1000 nM SNa for 24 h.

### RNA extraction and Illumina sequencing

Total RNA was extracted using the RNeasy Micro Kit (Qiagen) including an on-column DNase treatment to remove genomic DNA. The concentration of total RNA was determined using the Qubit RNA Assay Kit (Life Technologies). In order to evaluate the quality of the total RNA, RNA integrity number (RIN) was assessed using Agilent RNA 600 Nano Reagents and RNA Nano Chips in Agilent 2100 Bioanalyzer (Agilent Technologies). Sequencing of 10 RGC cultures was performed by MR DNA (www.mrdnalab.com) following the manufacturer’s guidelines (Illumina HiSeq) for paired end sequencing (151 bp). Using oligo(dT) magnetic beads, RNA with poly(A) tails were purified and fragmented into shorter sequences that were used as templates for cDNA synthesis. A total of 10 cDNA libraries were constructed using random-hexamer primers from 1 μg of total RNA from each sample using TruSeq RNA LT Sample Preparation Kits (Illumina). Following the library preparation, the final concentration of the library was measured using the Qubit dsDNA HS Assay Kit (Life Technologies) and the average library size was determined using the Aligent 2100 Bioanalyzer (Aligent Technologies). A total of 5 pM library was clustered using cBot (Illumina) and sequenced paired end for 300 cycles using the HiSeq. 2500 system (Illumina).

### *De novo* transcriptome assembly and annotation

Both *de novo* assembly and annotation were performed under contract by Genotypic Technologies (http://www.genotypic.co.in). The Illumina paired end raw reads were quality checked using FastQC (http://www.bioinformatics.babraham.ac.uk /projects/fastqc/). Illumina raw reads were processed by GT proprietary tools for adapters and low quality bases trimming towards the 3′-end. Only high-quality reads were considered for further downstream analysis. Biological replicates were pooled together to generate a reference transcriptome using Trinity^39^ using all default parameters with a k-mer of 25. Trinity partitions the sequence data into individual *de Bruijn* graphs, each representing the transcriptional complexity at a given gene or locus, and then processes each graph independently to extract full-length splicing isoforms and to identify transcripts derived from paralogous genes. Once assembled, the sequences were clustered based on similarity between sequences with CD-Hit v4.5.4^40^ using the following parameters: 95% coverage and 90% identity, -r 1, and others the default setting. Clustering reduces overall size of the database without removing any sequence information by only removing redundant or highly similar sequences. The longest sequences for each cluster was used as the representative sequence. The clustered transcripts with ≥300 bp in length were considered for the analysis. Transcripts were annotated using the Basic Local Alignment Search Tool (BLAST + 2.2.29) (E-value < 1.0e-4) using *Chordata* protein sequences from the Uniprot database^41^. The remaining unannotated transcripts were further annotated using Pfam database. After annotation, gene ontology terms were assigned using protein annotation through evolutionary relationships (PANTHER) in order to classify all transcripts identified in RGC cultures^42^. Gene ontology categories for biological processes, molecular functions, cellular components, protein classes, and pathways were used to identify the distribution of genes within each gene ontology category in RGC cultures.

### Differential gene expression analysis

The differential gene expression (DGE) levels between control (n = 3 cultures) and 1000 nM SNa exposure (n = 3) in RGCs were calculated using the DESeq method^43^. Briefly, after the variance was calculated for each gene, DGE was calculated assuming a negative binomial distribution for the expression level and a Fisher’s exact test was used to calculate P-value to test for significance between groups for each gene. Once the DGE was calculated, results were separated as up-regulated and down-regulated based on a |log2| > 0.5 cut off. A false discovery rate (FDR) at 5% was used for multiple hypothesis testing. All data have been deposited into the Gene Expression Omnibus (GSE106101), National Center for Biotechnology Information.

### qRT-PCR validation of differentially expressed genes

Samples used for qRT-PCR validation were obtained following the same cell culture methods that were used for RNA-Seq (i.e., passage 3 RGCs). The qRT-PCR was conducted using the SYBR green detection system to determine relative gene expression and to validate the DGE analysis. Primers were designed using Primer3^44^ and synthesized by Integrated DNA Technologies (Supplementary File S1). Primer sets were tested for specificity by subjecting the qRT-PCR products to a 1% agarose containing SYBR Safe DNA gel stain (Invitrogen) to ensure a single product was produced from each reaction. Each product was extracted from the gel using NucleoSpin Gel and PCR Clean-up kit (Macherey-Nagel) and sequenced to confirm primer specificity by StemCore Laboratories at the Ottawa Hospital Research Institute. The cDNA was prepared from 1 μg of total RNA using the Maxima First Strand cDNA Synthesis Kit for qRT-PCR (Thermo Scientific). The qRT-PCR analyses were conducted using the Maxima SYBR green qPCR Master Mix (Thermo Scientific) and CFX96 Real-Time PCR Detection System (Bio-Rad) to amplify the genes of interest. The thermal cycling parameters were: a single cycle Taq activation step at 95 °C for 3 min, followed by 40 cycles of 95 °C denaturation step for 10 s and one primer annealing temperature (56–63 °C) for 30 s depending on the primer set used. After 40 cycles, a melt curve was performed over a range of 65–95 °C with increments of 0.5 °C to ensure a single amplified product. Data were analyzed using CFX Manager Software package (Bio-Rad). The efficiency of each assay was 100 ± 10% and the R^2^ of each standard curve was >0.98. Relative mRNA abundance was calculated using the relative standard curve method based on Cq values and normalized using the NORMA-GENE algorithm^45^. Fold-change was calculated against the average of the control using normalized data. Fold-change for each group were presented as mean + SEM from 4 biological replicates (n = 4) assayed in duplicate. Data were assessed for normality with Shapiro-Wilk’s *W* test. A Student’s *t*-test was used to test for differences in mRNA levels between groups.

### Gene set enrichment analysis and sub-network enrichment analysis

Gene set and sub-network enrichment analyses were conducted using Pathway Studio 9.0 (Elsevier). Official gene symbols were used to map goldfish genes into Pathway Studio. A total of 28,597 genes were mapped in Pathway Studio and for duplicated gene symbols in the dataset, the gene that showed the “best p-value, highest magnitude fold change” was used for analyses. For gene set enrichment analysis (GSEA), genes were permutated 1000 times using the Kolmogorov-Smirnov classic approach as an enrichment algorithm. The gene set categories examined for enrichment included curated cell processes, cell signaling and receptor signaling pathways. Sub-network enrichment analysis (SNEA) for cell processes was performed to identify gene networks regulated in RGCs following SNa exposure. Utilizing known relationships (i.e., based on expression, binding, and common pathways) between genes, SNEA builds networks focused around gene hubs. For both GSEA and SNEA the enrichment P-value was set at P < 0.05. These analyses have been successfully used to build gene networks in teleost fish^46,47^.

## Results

### *De novo* transcriptome assembly and quality analysis

Before *de novo* transcriptome assembly, paired-end raw reads were processed to remove adapter fragments and low quality bases generating 161,704,300 clean reads from 163,641,713 raw reads. High-quality reads were subsequently assembled *de novo* using the Trinity program because there is no goldfish reference genome. Trinity assembler, with a *k*-mer size of 25 generated 170,967 unigenes with a total sequence length of 150,361,946 bp. Resulting sequences were binned into a non-redundant set of gene-oriented clusters or “unigene” clusters. Each cluster contains sequences that represent a unique gene. Lengths of the clusters ranged from 301 to 20,585 bp with a mean size of 879.5 and an N50 of 1318 bp. The N50 is the contig length such that of equal to or longer that constitutes half of the entire assembly. Among the clusters, 85,984 (47.7%) were 300–499 bp in length, 45,405 (27.4%) were 500–999 bp, 39,523 (24.8%) were 1,000–9,999 bp, and 55 (0.03%) were >10,000 bp in length.

### Annotation and gene ontology classification

**Figure 1:**
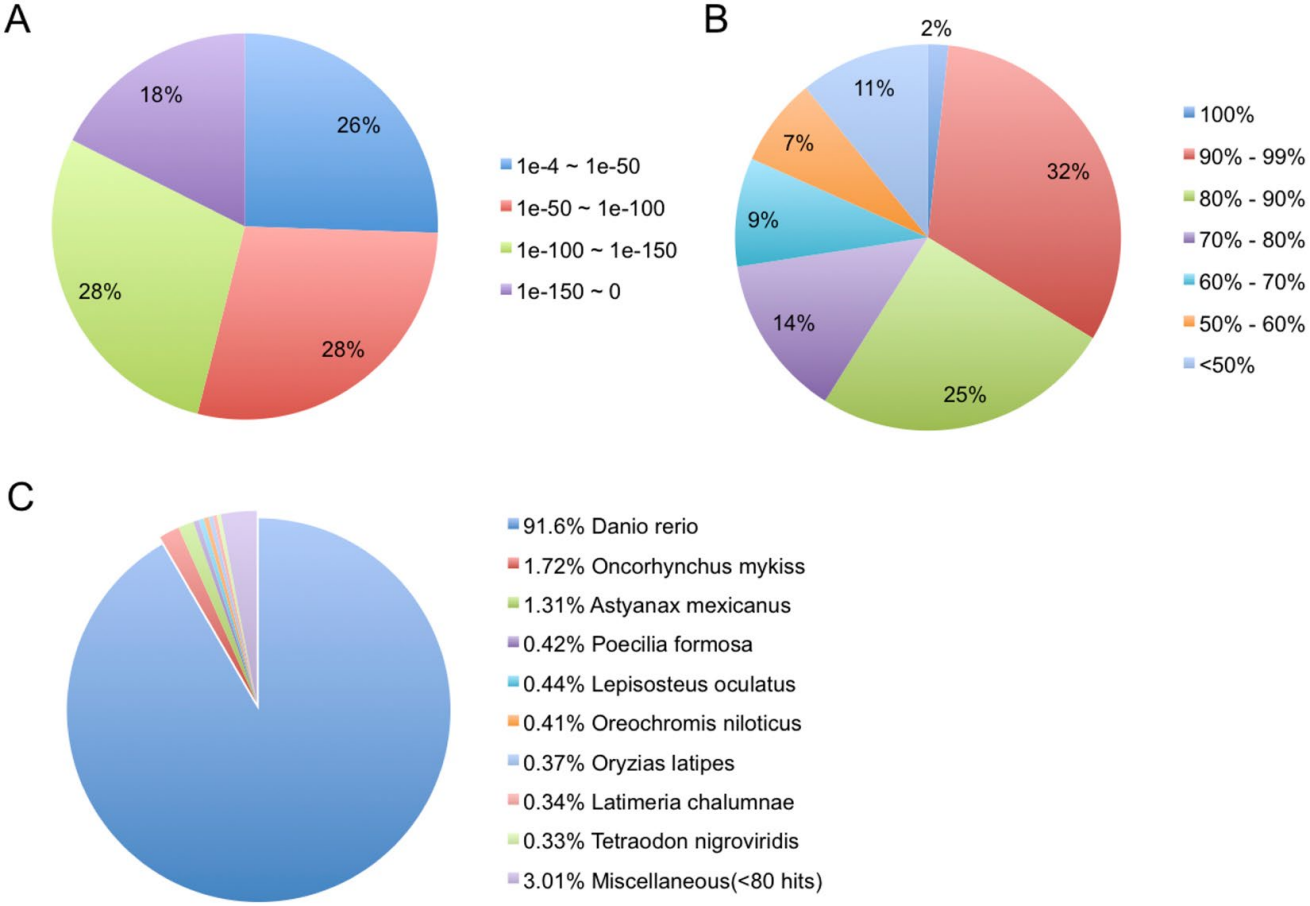
Annotation summary of assembled *Carassius auratus* RGC genes against the UniProt database. (**A**) E-value distribution of BLASTX hits for each transcript with an E-value cut off of 1e-4. (**B**) Similarity distribution of BLATX hits for each gene. (**C**) Distribution of the top BLASTX species hits in the UniProt database.

Annotation of the RGC transcriptome was performed by aligning transcripts ≥300 bp in length using BLASTX^41^ to all available *Chordata* protein sequences from UniProt database (using a cut off at E-value < 1.0e-4). The remaining unannotated transcripts were then compared to the Pfam database. A total of 67,486 unigenes were annotated (38.05% from UniProt and 1.42% from Pfam) while the remaining 96,145 unigenes (56.24% of all unigenes) could not be annotated, having no significant sequence similarity to any database entries (unknown function). This can be attributed to the lack of genomic information for goldfish, limiting identification of some of the transcripts. Overall, 59% of the transcripts were mapped with >80% identity and 74% of the matched sequence exhibited strong sequence similarity with an E-value < 1e-50 with respect to the E-value distribution pattern (Fig. 1A,B). The BLASTX top-hit species matched in the UniProt database were mostly other fish species, with the top three species being *Danio rerio* (91.6%), *Oncorhynchus mykiss* (1.72%), and *Astyanax mexicanus* (1.31%) (Fig. 1C).

**Figure 2:**
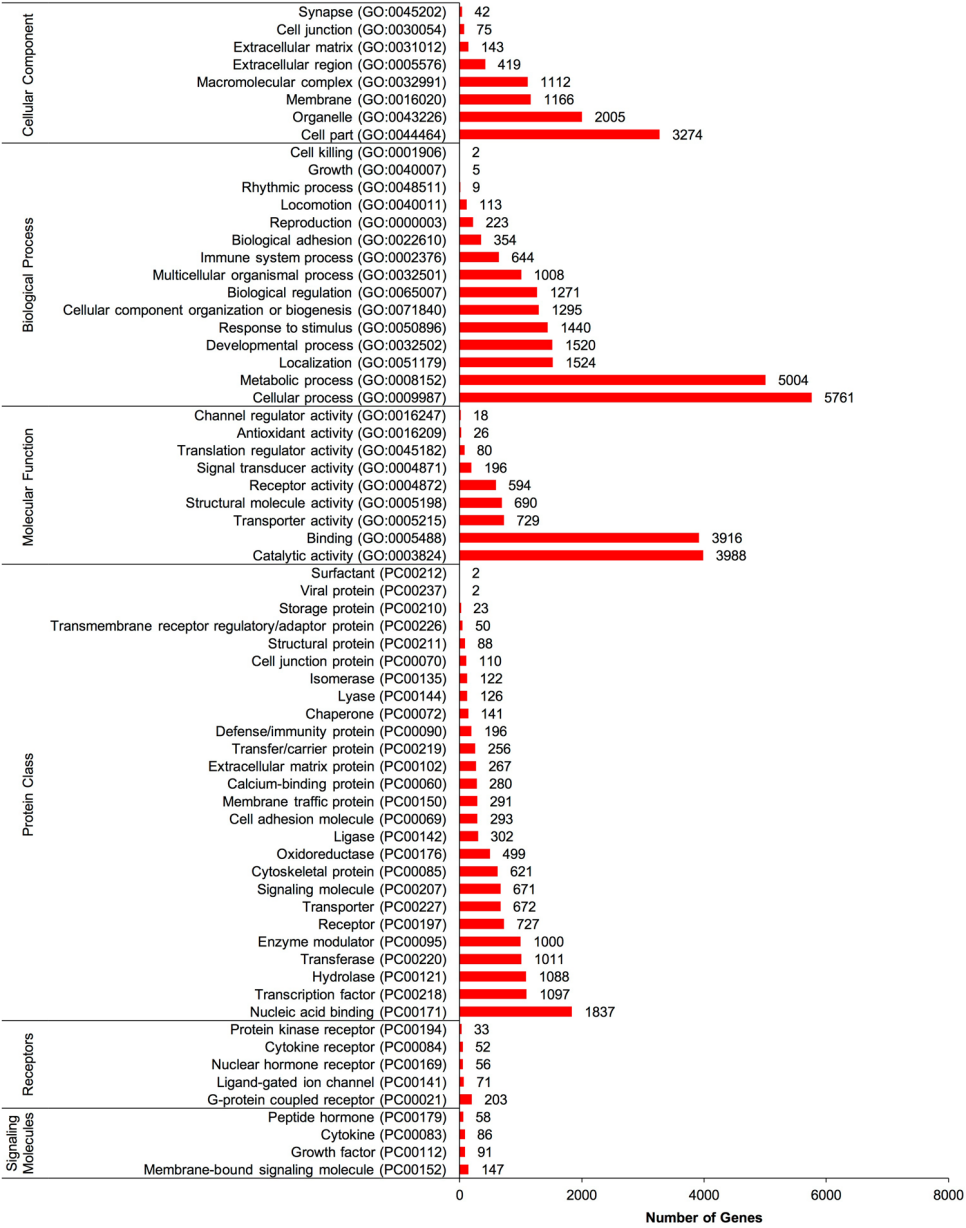
Gene ontology (GO) classification of assembled *Carassius auratus* RGC genes into molecular function, biological function, cellular component, protein class, receptor and signaling molecule categories. The number of genes ascribed to each classification is provided along with GO or protein class (PC) accession number.

The 17,620 non-redundant genes were assigned gene ontology (GO) terms for functional characterization. These annotated genes were classified in three main ontologies: cellular component (8,236 genes), biological process (20,173), and molecular function (10,237) (Fig. 2). Within the cellular compartment category, the RGC transcriptome was enriched in the cell part (3,274 genes; 39.8%), organelle (2,005; 24.3%), and membrane (1,166; 14.2%). In addition, a few genes were assigned to GO categories such as cell junction (75 genes; 0.9%) and synapse (42; 0.5%). In the biological process category, cellular process (5,761; 28.6%), metabolic process (5,004 genes; 24.8%), and localization (1,524; 7.6%) were predominant, suggesting these genes are involved in metabolism and cell growth. Other notable biological function allocations included transcripts involved in immune system processes (644 genes; 3.2%) and biological adhesion (354; 1.8%). Regarding molecular function, a high proportion of genes were assigned to catalytic activity (3,988, 39.0%), binding (3,916; 38.3%), and nucleic acid binding transporter activity (729; 7.1%).

The transcriptome was also categorized by protein class ontologies in the online program Panther (Fig. 2). Transcripts were classified as nucleic acid binding proteins (1,837 genes; 15.6%), transcription factors (1,097 genes; 9.3%), and hydrolases (1088 genes; 9.2%). Within protein classes, the receptor and signaling molecule ontologies were investigated (Fig. 2). The RGC transcriptome expresses an array of receptor classes such as G-protein coupled receptors (GPCRs) (203 genes; 48.9%), ligand-gated ion channels (71 genes; 10.1%), nuclear hormone receptors (56 genes; 13.5%), cytokine receptors (52 genes; 12.5%), and protein kinase receptors (3 genes 3; 8.0%). Within the signaling molecule category, genes were cataloged into membrane-bound signaling molecules (140 genes; 38.5%), growth factors (91 genes; 23.8%), cytokines (86 genes; 22.5%), and peptide hormones (58 genes; 15.2%) (Fig. 2). These GO analyses reveal a diverse receptor and signaling molecule profile, suggesting that RGCs can respond to and synthesize an array of hormones, peptides, cytokines, and growth factors.

Overall, a total of 5,344 annotated transcripts were assigned to 162 unique pathway ontologies. Based on the transcripts detected, the Wnt signaling pathway (239 genes; 4.6%) was enriched, as well as gonadotropin-releasing hormone receptor pathway (200 genes; 3.8%), chemokine and cytokine signaling pathways (188 genes; 3.6%), and angiogenesis (170 genes; 3.3%) (Table 1). When considering all pathway ontologies, it was noted that many were neurotransmitter receptor-related such as serotonin (PANTHER pathway accession numbers: P04374, P04373, P04376, P04375), dopamine (P05912), acetylcholine (P00044, P00043, P00042), GABA (P05731), glutamate (P00037, P00039, P00040, P00041), adrenaline (P04378, P04377, P00002, P04379), and histamine (P04385, P04386). A number of genes were assigned to hormone related pathways, including gonadotropin-releasing hormone receptor signaling (P06664), oxytocin receptor signaling (P04391), thyrotropin-releasing hormone receptor signaling (P04394), corticotropin-releasing factor receptor signaling (P04380), opioid proopiomelanocortin pathway (P05917), vasopressin synthesis (P04395), cholesterol biosynthesis (P00014), and androgen/estrogen/progesterone biosynthesis (P02727). In addition, several immune system pathways were identified, for example chemokine and cytokine signaling (P00031), epidermal growth factor signaling (P00018), fibroblast growth factor signaling (P00021), transforming growth factor β (P00052), interleukin signaling (P00036), both T and B cell activation (P00053, P00010), toll receptor signaling (P00054), and interferon γ signaling (P00035). A full list of all pathways, receptors, signaling molecules and other gene ontology classifications can be found in Supplemental File S2.

**Table 1:** Top 25 assigned pathway ontologies for assembled *Carassius auratus* RGC genes based on the number of genes identified in each pathway.

Pathway Name	Pathway Accession	# of genes identified
Wnt signaling pathway	P00057	239
Gonadotropin releasing hormone receptor pathway	P06664	200
Inflammation mediated by chemokine and cytokine signaling pathway	P00031	188
Angiogenesis	P00005	170
Integrin signaling pathway	P00034	166
CCKR signaling map	P06959	164
EGF receptor signaling pathway	P00018	132
PDGF signaling pathway	P00047	128
FGF signaling pathway	P00021	118
Huntington disease	P00029	110
Heterotrimeric G-protein signaling pathway-Gi alpha and Gs alpha mediated pathway	P00026	108
Cadherin signaling pathway	P00012	105
Alzheimer disease-presenilin pathway	P00004	101
Apoptosis signaling pathway	P00006	99
TGF-beta signaling pathway	P00052	93
Parkinson disease	P00049	93
Heterotrimeric G-protein signaling pathway-Gq alpha and Go alpha mediated pathway	P00027	85
Ras Pathway	P04393	82
Endothelin signaling pathway	P00019	74
T cell activation	P00053	73
Interleukin signaling pathway	P00036	72
p53 pathway	P00059	71
B cell activation	P00010	70
Cytoskeletal regulation by Rho GTPase	P00016	68
VEGF signaling pathway	P00056	66

### Differential gene expression analysis following SNa treatment

With a fold-change cut off |log2| > 0.5, the DGE analysis revealed 1,776 differentially expressed genes (DEGs) after SNa treatment at P < 0.05 and 123 DEGs at P < 0.001. A total of 42 DEGs, 31 up-regulated and 11 down-regulated, passed the false discovery rate (FDR; < 0.05) correction for multiple hypothesis testing. All of the 31 up-regulated DEGs could not be annotated at this time, thus likely representing novel transcripts with no detected similarities to other species. Of the 11 down-regulated DEGs in goldfish RGCs, 7 were annotated (Table 2). Transcripts that were differentially down-regulated (FDR < 0.05) genes included: mothers against decapentaplegic homolog 6 (*smad6b*), fibroblastic growth factor 4 (*fgf4*), NGFI-A binding protein 1a (*nab1a*), BAI1-associated protein 2b (*baiap2b*), GRB2-related adaptor protein a (*grapa*), phosphodiesterase 8 A (*pde8a*), tRNA nucleotidyl transferase (*trnt1*), and heat shock protein beta 11 (*hspb11*).

**Table 2:** List of differentially expressed genes compared to control in *Carassius auratus* RGC culture after 24 h 1000 nM SNa treatment (FDR < 0.05).

**Gene symbol**	**Gene Name**	**Uniprot Accession**	**Fold Change**	**P-Value**	**FDR Adjusted P-Value**
*smad6b*	Mothers against decapentaplegic homolog 6	tr|Q1L8Y3|Q1L8Y3_DANRE	0.40	7.34E-11	2.80E-07
*fgf4*	Fibroblast growth factor 4	tr|Q9DFC9|Q9DFC9_DANRE	0.29	5.03E-10	1.78E-06
*nab1a*	NGFI-A binding protein 1a	tr|Q1LWI4|Q1LWI4_DANRE	0.28	9.87E-07	2.33E-03
*baiap2b*	BAI1-associated protein 2b	tr|U3JAA2|U3JAA2_DANRE	0.46	1.65E-06	3.63E-03
*grapa*	GRB2-related adaptor protein a	tr|Q503S8|Q503S8_DANRE	0.35	3.21E-06	6.43E-03
*pde8a*	Phosphodiesterase 8 A	tr|H9GZ87|H9GZ87_DANRE	0.31	1.25E-05	2.08E-02
*trnt1*	tRNA nucleotidyl transferase	tr|A7MCH7|A7MCH7_DANRE	0.08	1.30E-05	2.14E-02
*hspb11*	Heat shock protein beta 11	sp|A5JV83|HSPBB_DANRE	0.33	2.60E-05	3.88E-02

### qRT-PCR validation of DGE analysis

**Figure 3:**
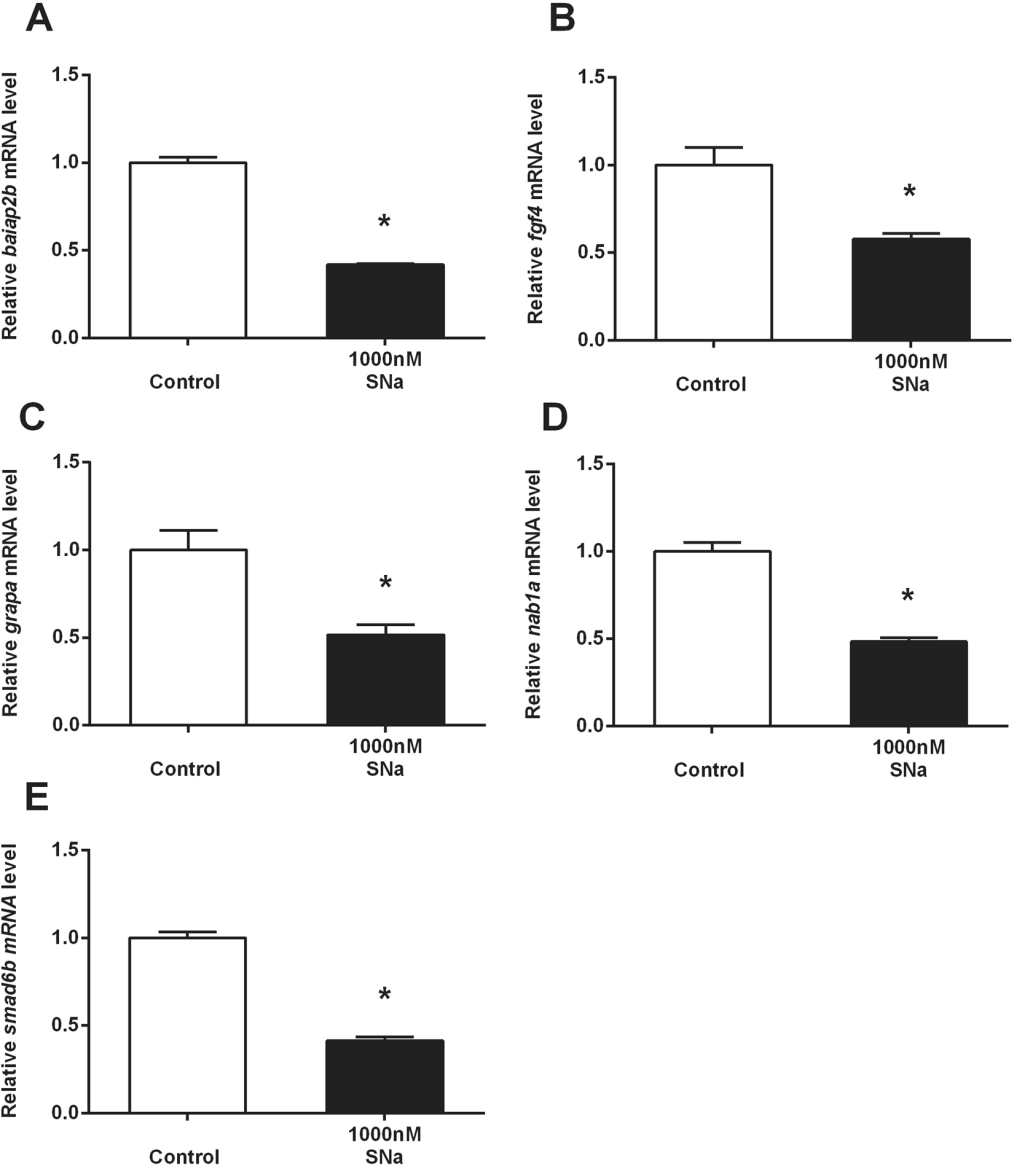
Quantitative real-time PCR analysis for (**A**) *baiap2b*, (**B**) *fgf4*, (**C**) *grapa*, (**D**) *nab1a* and (**E**) *smad6b* mRNA in *Carassius auratus* RGC culture exposed to 1000 nM SNa. Data were normalized and defined as fold-change relative to control. Bars represent the mean + SEM (n = 4). Treatment groups marked by asterisks have significantly different mRNA levels compared to control (P < 0.05).

In order to validate the expression of annotated DEGs identified by RNA-Seq, 5 primer pairs were designed based on the corresponding assembled sequence. The fold-changes quantified by RNA-Seq and qPCR, respectively for these 5 DEGs were: 0.46 and 0.42 (P < 0.05) for *baiap2b*, 0.29 and 0.58 (P < 0.05) for *fgf4*, 0.35 and 0.52 (P < 0.05) for *grapa*, 0.28 and 0.48 (P < 0.05) for *nab1a*, and 0.40 and 0.41(P < 0.05) for *smad6b* (Fig. 3). Overall, the expression profiles obtained by RNA-Seq analysis and qPCR were highly comparable in the direction of change.

### Gene set enrichment analysis

GSEA revealed a total of 17 pathways that were statistically significant (P < 0.05) and had a median fold change >5% (Table 3). Pathways were grouped by three categories: cell processes (4 pathways), cell signaling (2 pathways), and receptor signaling (11 pathways). Cell processes involved in adherens and tight junction assembly such as nectins, claudins, cadherins, and junctional adhesion molecules (JAMs) were all significantly increased (10–17% or 1.10- to 1.17-fold) indicating that SNa up-regulates gene networks involved in cell-cell junctions. GSEA for cell signaling pathways revealed that actin cytoskeleton regulation and T cell activation pathways were significantly increased in SNa-treated RGCs. SNa significantly increased genes involved in four different T-cell receptor signaling pathways. Besides the T-cell receptor, other receptors such as glucagon receptor, dopamine D1 receptor, tumor necrosis factor receptor (TNFR), tumor necrosis factor receptor superfamily member 1 A (TNFSF1A), and neural cell adhesion molecule I (NCAMI) all shared similar CREB/ELK-SRF signaling. In addition, interleukin 6 receptor (ILR) and ephrin receptor (ephrinR) signaling pathways were also responsive to SNa treatment. A complete list of all enriched pathways is provided in Supplementary File S3.

**Table 3:** Gene set enrichment analysis (GSEA) for transcripts in *Carassius auratus* RGC cultures treated with 1000 nM SN (P < 0.05).

**Gene Set Category**		**Total # of entities**	**# of measured entities**	**Median change**	**P-value**
Ariadne Cell Process Pathways	Adherens Junction Assembly (Nectin)	104	57	1.10	0.045
	Tight Junction Assembly (Claudins)	137	62	1.13	0.022
	Adherens Junction Assembly (Cadherins)	133	53	1.14	0.016
	Tight Junction Assembly (JAMs)	119	55	1.17	0.024
Ariadne Cell Signaling Pathways	Actin Cytoskeleton Regulation	551	357	1.10	0.009
	T Cell Activation	957	490	1.11	0.003
Ariadne Receptor Signaling Pathways	IL6R - > STAT signaling	8	8	1.08	0.047
	EphrinR - > actin signaling	216	143	1.12	0.020
	T-cell receptor - > NF-kB signaling	176	33	1.15	0.000
	T-cell receptor - > AP-1 signaling	180	33	1.17	0.025
	T-cell receptor - > CREBBP signaling	176	25	1.19	0.027
	GlucagonR - > CREB/ELK-SRF/SP1 signaling	42	27	1.20	0.040
	DopamineR1 - > CREB/ELK-SRF signaling	40	25	1.20	0.016
	T-cell receptor - > ATF/CREB signaling	195	45	1.21	0.021
	TNFR - > CREB/ELK-SRF signaling	45	30	1.23	0.050
	TNFRSF1A - > CREB/ELK-SRF signaling	41	32	1.23	0.014
	NCAM1 - > CREB/ELK-SRF/MYC signaling	27	21	1.26	0.046

Only those pathways with a median fold-change greater than 5% are presented. The number of measured entities in the pathway is reported along with the number of measured entities and median fold-change for the pathway.

### Sub-network enrichment analysis

**Figure 4:**
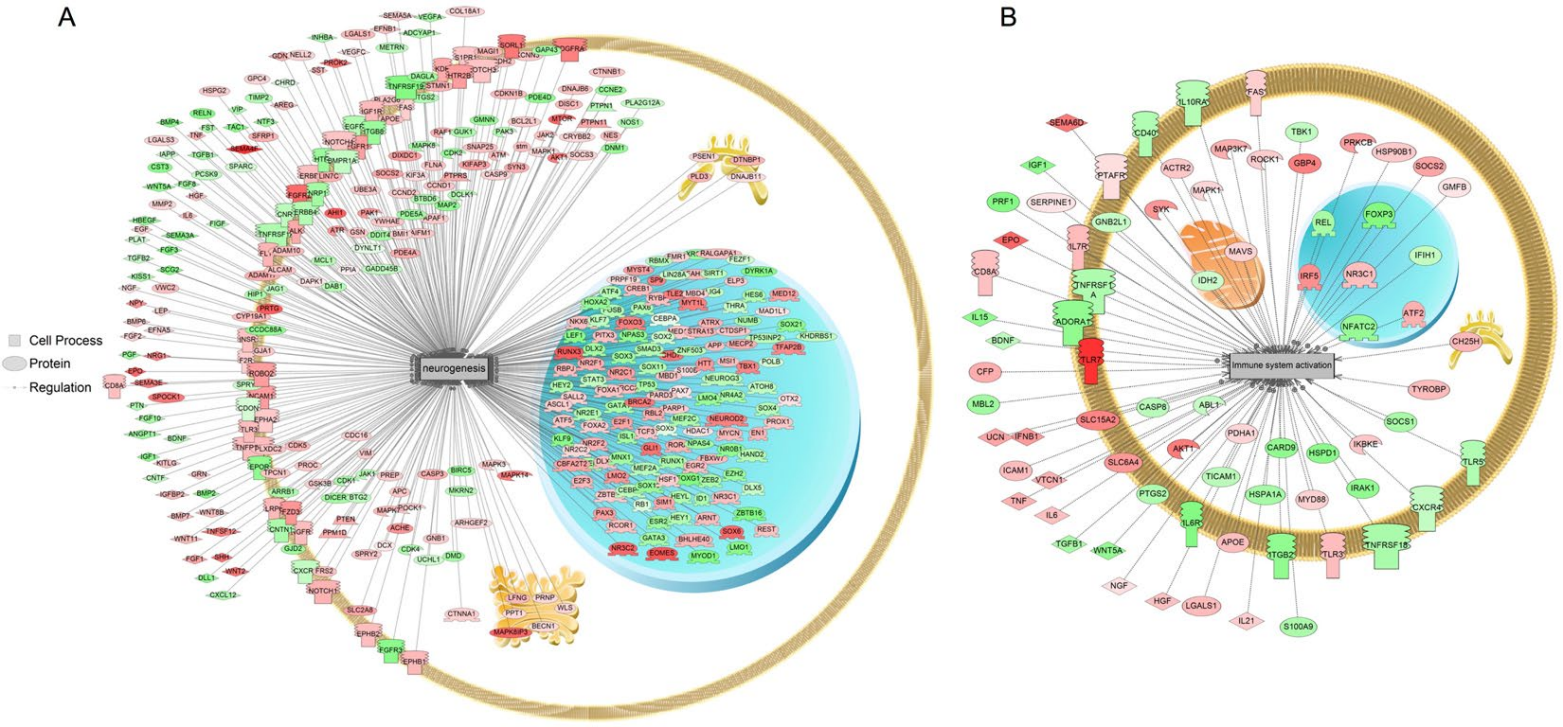
Subnetwork enrichment analysis (SNEA) indicated that genes involved in (**A**) neurogenesis and (**B**) immune system activation were significantly enriched in *Carassius auratus* RGC cultures following 24 h exposure to 1000 nM SNa treatment (P < 0.05). Red indicates that gene expression was increased and green indicates that gene expression was decreased. All abbreviations are provided in Supplemental File S4.

Using SNEA, a total of 192 cellular processes were significantly responsive to SNa exposure. Sub-networks that included processes related to CNS and immune system were major biological themes regulated by SNa (Table 4). All 19 sub-networks involved in processes related to the CNS were increased (7–70% or 1.07- to 1.70-fold). Examples of these sub-networks included neurogenesis, glial cell development, neuronal activity, synaptic plasticity, axon guidance and extension. A total of 19 sub-networks were associated with cellular processes of the immune system indicating that this is another major theme in the transcriptomic response to SNa. There were 18 sub-networks that were increased (5–53% or 1.05- to 1.53-fold) and 1 sub-network that was decreased (−13% or −1.13 fold), indicating that SNa has a largely stimulatory effect on diverse processes in RGCs. These processes included immune system activation, immunoreactivity, lymphocyte activation, macrophage response leukocyte function and differentiation. Neurogenesis (Fig. 4A) and immune system activation (Fig. 4B) are prominent examples of sub-networks that were significantly regulated by SNa. All identified sub-networks are presented in Supplementary File S3.

**Table 4:** Cell processes identified by sub-network enrichment analysis (SNEA) that were regulated by 1000 nM SNa in primary *Carassius auratus* RGC cultures.

	Gene Set Seed	Total # in pathway	# of Measured Neighbors	Median change	P-value
Processes related to CNS	Axon guidance	210	160	1.17	0
	Transmission of nerve impulse	598	321	1.1	0
	Cognition	212	126	1.11	0.004
	Innervation	172	113	1.08	0.005
	Brain function	150	89	1.14	0.006
	Cerebellum development	14	8	1.44	0.007
	Neuronal activity	263	133	1.06	0.008
	Neuroproliferation	6	5	1.7	0.01
	Hindbrain development	15	11	1.44	0.013
	Synaptic transmission	509	288	1.13	0.013
	Synaptic plasticity	454	284	1.1	0.013
	Schwann cell formation	15	12	1.26	0.016
	Memory	721	385	1.08	0.016
	Neuron development	103	69	1.1	0.029
	Dentate gyrus development	6	6	1.34	0.03
	Neurogenesis	561	383	1.11	0.031
	Central nervous system function	25	15	1.26	0.041
	Axon extension	74	59	1.16	0.042
	Glial cell development	17	11	1.25	0.044
Processes related to immunity	B-cell activation	233	105	1.1	0.001
	Leukocyte function	158	74	1.09	0.003
	Granulocyte adhesion	52	27	1.11	0.009
	Peritoneal macrophage function	10	5	1.34	0.014
	Immunoreactivity	463	260	1.13	0.014
	Eosinophil degranulation	43	15	1.31	0.015
	T-cell homeostases	100	50	1.05	0.016
	Respiratory burst	185	92	1.1	0.017
	NK cell mediated cytotoxicity	367	133	1.09	0.018
	Leukocyte accumulation	84	43	1.1	0.019
	Leukocyte differentiation	9	5	1.53	0.022
	B lymphocyte proliferation	277	148	1.07	0.022
	Lymphocyte activation	277	131	1.11	0.023
	Lymphocyte aggregation	10	6	1.32	0.027
	Macrophage response	48	19	1.1	0.028
	Immune system activation	163	76	1.07	0.033
	Leukocyte tethering / rolling	106	58	1.11	0.041
	Phagocyte activity	167	80	1.1	0.046
	Granulosa cell differentiation	46	34	−1.13	0.047

Only those sub-networks with a median fold-change greater than 5% in the cell process are presented (P < 0.05). Reported here are only some major cell processes affected by SNa in RGCs.

## Discussion

Given what has been reported previously about teleost RGCs^13,48^, we predicted that transcriptomic profiling would identify genes related to steroidogenesis and neurogenesis, in addition to several receptors for classical neurotransmitters. Goldfish RGCs do indeed express genes related to these key processes, but also express a much wider range of cell surface and nuclear receptors, including numerous neurogenesis- and immune-related genes. A total of 17,620 non-redundant unigene clusters were identified by RNA sequencing and GO analysis revealed a diverse receptor and signaling molecule profile. This suggests that teleost RGCs can respond to and synthesize an array of hormones, peptides, cytokines, and growth factors.

### A reference transcriptome for cultured teleost RGCs

We extend our observations on the expression of glial fibrillary acidic protein (*gfap*), brain fatty acid binding protein (*blbp*), aromatase B (*cyp191b*), and related proteins^16^ to transcriptomic characterization of goldfish RGCs. Further validating this culture system, several critical markers previously reported to be expressed in zebrafish RGCs in the adult telencephalon and diencephalon^13^ were identified by RNA-Seq in goldfish RGCs. These include the well-documented *cyp191b*, *blbp*, *gfap*, and also calcium binding protein β (*s100b*), connexin 43 (*cx43*), glial high-affinity glutamate transporter (*slc1a3a*), glutamine synthase (*glula*), nestin (*nes*), sex determining region Y box 2 (*sox2*), and vimentin (*vim*). These glial markers have been used to characterize RGCs in zebrafish and provide the cell with the abilities of neurotransmitter uptake (*slc1a3a, glula*), estrogen production (*cyp19a1b*), and generation of calcium waves (*cx32*)^13^. Identification of several steroidogenic enzyme transcripts such as steroidogenic acute regulatory protein (*star*), 17α-hydroxylase (*cyp17a1*), 17β-hydroxysteroid dehydrogenase (*hsd17b10*), aromatase B (*cyp19a1b*), and 5α-reductase (*srd5a1*), suggest that these cells are capable of *de novo* estrogen production from cholesterol and are able to produce pregnenolone and progesterone intermediates, confirming previous observations that these are steroidogenic cells^48^. Here we also show that cultured goldfish RGCs express progesterone receptor (*pgr*), androgen receptor (*ar*), G-protein coupled estrogen receptor (*gper1*), estrogen receptor α (*esr1*), estrogen receptor β1 (*esr2b*), and estrogen receptor β2 (*esr2a*). These data support other reports in zebrafish that *in vivo* RGCs express Pgr protein^49^ as well as observations for the distribution of *ar*, *esr1, esr2a*, and *esr2b* mRNAs with aromatase B expression in RGCs^50^. The expression of nuclear estrogen receptor mRNAs has been documented before in goldfish RGC cultures^17^; however, to the best of our knowledge, this is the first report demonstrating that *gper1* is expressed by RGCs. The combination of numerous steroidogenic enzymes and steroid receptors expressed by RGCs strongly support the hypothesis that RGCs are both a source and target of neurosteroids^50^.

### RGCs express multiple receptors

We report that RGCs express receptors for various neurotransmitters including 5-HT (1 A, 2B, 2 C, 6), DA (D2, D4), GABA (GABA_A_, GABA_B_), acetylcholine (M4, M5), adrenaline (α2a, α2c, β1, β2), adenosine (A1, A2B), and histamine (H1, H3). Our previous studies showed that DA D1 receptor activation in RGCs can increase aromatase B and regulate protein networks associated with progenitor cell functions^16,18^. Furthermore, RGCs may also be under the direct control of 5-HT, as 5-HT neurons share a close distribution with RGCs in the zebrafish paraventricular organ and *in vivo* inhibition of 5-HT synthesis decreases RGC proliferation in zebrafish^19^. In support of our RNA-Seq data, goldfish RGCs have previously been documented to express adenosine A1 receptors^51^ and activation of this receptor in rabbit RGCs causes ATP-evoked increases in calcium that regulate proliferation^52^. In mice, RGC fate is under the control of GABA excitation where high levels of activation result in quiescence, while low levels of excitation cause either symmetrical or asymmetrical cell divisions^53^. Histamine H1, H2, and H3 receptors are expressed by mammalian astrocytes and regulate astrocytic functions such as calcium influx, energy metabolism, neurotransmitter clearance, neurotrophic activity, and immune response^54^. Together, this panel of expressed neurotransmitter receptors suggest that goldfish RGCs are in communication with numerous neuronal subtypes. Goldfish RGCs also express receptors for growth hormone (*ghra*), growth hormone-releasing hormone (*ghrh*), thyroid hormone (*thraa, thrab*, *thrb*), thyrotropin releasing-hormone (*trhr2*), prolactin-releasing peptide (*prlhr2a*), and prolactin (*prlrb*), amongst others. Few studies have investigated hormonal control of glial cells. Best described are the stimulatory effects of thyroid hormones on neural progenitor proliferation in mammals^55,56^ and estrogenic stimulation of aromatase B expression in zebrafish RGCs^57^. We have found that both thyroid hormone and estrogen receptors are expressed in female goldfish RGCs in our cultures. The response of these cells to thyroid hormones has not been investigated, but we have shown previously that goldfish RGCs respond directly to estradiol^17^. These new transcriptomic data reported here are required to fully investigate hormonal regulation of this progenitor cell type in the CNS.

### RGCs express multiple immune system genes

The GO analysis also identified the presence of many immune system pathways, cytokines, and cytokine receptors in RGCs, indicating that fish RGCs may have important functions in brain immune reactivity. In fish brain, RGCs are the main macroglia due to the lack of typical stellate astrocytes^9^, thus RGCs likely share some functions with mammalian astrocytes in order to maintain brain homeostasis and to regulate neuroinflammation^58–60^. The transcriptome of goldfish RGCs reveal key proinflammatory molecules and receptors, including interleukin/interleukin receptors, toll-like receptors, tumor necrosis factor/tumor necrosis factor receptors, and transforming-growth factor β (TGFβ). Although their roles in neuroinflammation are less established than those in astrocytes, RGCs in fish brain respond to inflammatory cues to initiate the regenerative response. Unlike mammals, in which brain injury and neuroinflammation can result in glial scarring that obstructs neurogenesis^61^, neuroinflammation in fish directly activates RGC proliferation and subsequent neurogenesis through leukotriene C4 signaling^62^. In addition, the expression of chemokine/chemokine receptors implicates RGCs in the control of processes such as neural migration, axonal guidance, and neural regeneration that involve chemotaxis^63–65^. Zebrafish RGCs express the chemokine Cxcl2 and its receptor Cxcr4^65^ and also the chemokine receptor Cxcr5, which regulates RGC proliferation and differentiation^66^. Thus, the immune system appears to be a prominent functional theme based this transcriptomic study.

### The secretogranin-derived peptide SNa regulates multiple functions in cultured goldfish RGCs

The close neuroanatomical relationship between abundant RGCs and soma of SNa-immunoreactive neurons in the preoptic nucleus of female goldfish^20^ led us to postulate that SNa is a regulator of RGC physiology. It is known that these preoptic SNa-positive cells are the magnocellular and parvocellular neurons expressing the nonapeptides isotocin and vasotocin^67,68^, the fish homologs of mammalian oxytocin and vasopressin, respectively. Our data have identified the expression of both oxytocin (*oxtr*), and vasopressin (*avpr2l*) in RGCs providing further support for potential interactions between these neuropetidergic neurons and RGCs.

We report that SNa decreased the expression of *smad6b, nab1a*, and *fgf4* in cultured goldfish. The mammalian homologue Smad6 is a negative regulator of bone morphogenic proteins^69^ and this gene has been linked to the regulation of stem/progenitor cell behaviours^70^. Our study showed that SNa decreased *nab1a* mRNA, the orthologue to mammalian *nab1*. Nerve growth factor-induced gene A binding protein 1 (NAB1; also called EGR1 binding protein 1) is an active corepressor that negatively regulates the transcriptional activity of nerve growth factor-induced gene A *(NGFI-A*)^71^. Furthermore, NGFI-A is an immediate-early gene and is responsive to various extracellular stimuli such as growth factors, hormones, and neurotransmitters in order to regulate the growth, proliferation, and differentiation of a variety of cell types^72^, including astrocyte growth and proliferation^73,74^. As a corepressor, NAB1 completely blocks transcription facilitated by NGFI-A^71^. Therefore, SNa-induced decreases in NAB1A may modulate NGFI-A-dependent processes in RGCs. It was also shown here that SNa reduced the relative expression levels of *fgf4*. The Fgfs are polypeptides that regulate cell proliferation, differentiation, and migration in the developing and mature CNS^75^, including mammalian RGC self-renewal and differentiation^76,77^. These observations and our pathway analyses all support the proposal that SNa regulates networks implicated in glial cell development, neurogenesis and neuronal proliferation.

Many SNa-regulated transcripts in RGCs are related to cell-cell junctions and cell-cell communication. Transcripts involved in both tight (claudins, JAMs) and adherens (nectin, cadherins) junction assembly were significantly affected by SNa. Cell-cell adhesion molecules are important in neuronal cell migration, axon guidance, synapse formation, and in forming glial networks^78^. As SNa regulates genes involved in the assembly of adherens and tight junctions, this neuropeptide is thus implicated in regulating processes that involve glial-glial and/or glial-neuron interactions.

A major finding in this study is that multiple immune response pathways in RGCs were affected by SNa exposure. Gene set enrichment revealed that IL-6R signaling, TNFR signaling, T-cell activation, and several T-cell receptor signaling pathways were preferentially affected by SNa. It has been established that other neuropeptides such as vasoactive intestinal peptide^79^, substance P^80^, and somatostatin^81^ can affect IL-6 expression in astrocytes, and SNa may fall into this category. SNa also increased many immune system processes based upon the transcriptome profiles, some of which included immune system activation, phagocyte activity, leukocyte function, macrophage response, lymphocyte aggregation, and activation. Regulation of immune system responses has been previously reported for SN in other cell types. For example, after experimental autoimmune encephalomyelitis is induced in the rat brain, there is a close correlation between SN-immunoreactivity and macrophage infiltration, indicating that SN may play a role in leukocyte recruitment^82^. Both *in vivo* and *in vitro*, SN can stimulate the migration of human monocytes, also acting synergistically with other sensory neuropeptides like substance P or somatostatin^37^. SN has also been shown to be a chemoattractant for human eosinophils, comparable in activity to interleukin-8^83^ and SN can stimulate natural killer cell migration and cytokine release^84^. We propose that SNa may be acting as a proinflammatory regulator of goldfish RGCs, although further studies are needed to directly test this hypothesis.

A number of pathways related to CNS processes were regulated by SNa in cultured RGCs, demonstrating that SNa may have diverse functional roles in the brain. These included memory, neurogenesis, transmission of nerve impulses, synaptic transmission and plasticity, axon extension and guidance, and neuron and glial cell development among others. Overall, pathways in this general biological theme of “CNS-related” showed increased expression compared to untreated RGCs, suggesting that SNa has a largely stimulatory effect on these biological processes. RGCs are neuronal precursors in the adult fish CNS and are involved in the guidance of migrating young neurons^85^. Mammalian SN has been shown to act as a trophic peptide stimulating neurite outgrowth^27^ and can also activate the Jak2/Stat3 pathway to promote neuroprotection and enhance neurogenesis in murine models of stroke^28^. Perturbations in the gene networks identified here are consistent with documented neurogenic and neuroprotective effects of SN. It is tempting to speculate on the importance of SNa-regulated transcripts in RGCs that may be involved in some aspects of neurogenesis and synaptic plasticity. To the best of our knowledge, there are no comparable data available showing effects of SN on a glial cell type. Therefore, it will be necessary to specifically address this question in future research. Nevertheless, key regulators such as neurogenic differentiation factor 2^86^ and myelin transcription factor-like 1^87^ among others were expressed in goldfish RGCs and may be regulated by SNa.

## Conclusion

RGCs express an array of hormone, neurotransmitter, and neuropeptide receptors, suggesting a multiplicity of new functions critical to neuronal-glial communication. The identification of immune system pathways, proinflammatory signals and receptors indicate that RGCs may also be involved in regulating neuroinflammation processes in the fish brain. It will be important to determine the neuroanatomical localization of many of these genes to link them to *in vivo* functions. We show that exposure to the neuropeptide SNa modulated the expression of multiple genes in pathways associated with CNS and immune function. Several independent lines of evidence suggest that the yet unidentified SN receptor is G-protein coupled^88^. How key components of GPCR signaling pathways are linked to a putative membrane SN receptor in RGCs will be the focus of our future research. We hope that our annotated RGC transcriptome will provide a resource for future studies.

## Electronic supplementary material

Supplemental file S1

Supplemental file S2

Supplemental file S3

Supplemental file S4
