# Supplementary material for: Secretoneurin A regulates neurogenic and inflammatory transcriptional networks in goldfish (Carassius auratus) radial glia
Source: Sci Rep. 2017 Nov 2;7:14930. doi: 10.1038/s41598-017-14930-8 (PMC5668316; doi:10.1038/s41598-017-14930-8)
Supplement: Supplementary file 1 — Supplemental file S1 [file 41598_2017_14930_MOESM1_ESM.pdf]

Secretoneurin A regulates neurogenic and inflammatory transcriptional networks in goldfish (*Carassius auratus*) radial glia

Dillon F. Da Fonte, Christopher J. Martyniuk, Lei Xing, Adrian Pelin, Nicolas Corradi, Wei Hu, Vance L. Trudeau

Supplemental File S1

	Primer Sequence (Forward)	Primer Sequence (Reverse)
smad6b	CGGCGAATAAAATCCACAGA	CTGAACGGAACCAGGAACA
grapa	GGCTCACCCTTTCCTCTCTT	CCCTTCCTGTTCACCAACTC
fgf4	GGGCTACGCATTCCATTCT	TAGGTGTTTTGGGGGTCTTG
nab1a	CGAACACAGCCTATCTCCATC	TAGTCGCTCTACGCACTCCA
baiap2b	TCCGTCCATTTCTCTCCAAC	GACCTTCTCCAACACCCTACCT
